# Lipoma Arborescens of the Knee: Report of Three Cases and Review of the Literature

**DOI:** 10.1155/2017/3569512

**Published:** 2017-01-24

**Authors:** Ioannis Tsifountoudis, Dimitrios Kapoutsis, Anastasios-Nektarios Tzavellas, Ioannis Kalaitzoglou, Apostolos Tsikes, George Gkouvas

**Affiliations:** ^1^Department of Radiology, 424 General Military Hospital, Ring Road N. Efkarpias, 56403 Thessaloniki, Greece; ^2^Department of Orthopaedics, 424 General Military Hospital, Ring Road N. Efkarpias, 56403 Thessaloniki, Greece; ^3^Asclepios Diagnostic Center, 211 A. Papanastasiou Street, 54250 Thessaloniki, Greece

## Abstract

Lipoma arborescens is a chronic, slow-growing, intra-articular lesion of benign nature, which is characterized by villous proliferation of the synovium, with replacement of the subsynovial connective tissue by mature fat cells. It usually involves the suprapatellar pouch of the knee joint. It is not a neoplasm but is rather considered a nonspecific reactive response to chronic synovial irritation, due to either mechanical or inflammatory insults. We report three cases of lipoma arborescens affecting the knee, the first in a young male without previous history of arthritis or trauma, the second in a 58-year-old male associated with osteoarthritis, and the final in a 44-year-old male diagnosed with psoriatic arthritis, which cover the entire pathologic spectrum of this unusual entity. We highlight the clinical findings and imaging features, by emphasizing especially the role of MRI, in the differential diagnosis of other, more complex intra-articular masses.

## 1. Introduction

Synovium is an uncommon site for primary tumors and most of them are benign. Lipoma arborescens (LA) is one of them. It is a relatively unusual, benign, intra-articular lesion, which more often appears in the knee [1]. Other sites such as the shoulder, the elbow, the wrist, the hip, and the ankle may also be affected [2]. Bilateral involvement of the knees has also been described [3].

We report three patients with LA of the knee and their clinical appearance and symptoms, imaging, and histological characteristics, as well as their treatment and follow-up.

## 2. Case Presentations

### 2.1. Patient 1

A 30-year-old man, army officer, presented with a 2-year history of slowly progressive swelling of his right knee. The patient complained of night knee pain that woke him up. He also stated that while knee swelling was growing, pain was reducing. He did not report prior trauma, surgery, or infection in his knee.

On clinical examination, there was painless, diffuse swelling in the suprapatellar area. The knee had full range of motion and showed no signs of meniscal lesions, nor anteroposterior and collateral instability.

All laboratory studies were normal.

Plain radiographs depicted an area of dense soft tissue in the suprapatellar pouch. The femur and tibia were normal, with normal joint space.

**Figure 1:**
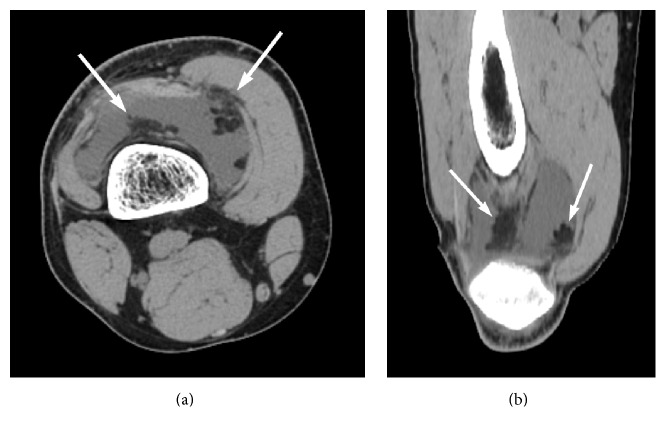
CT images on axial (a) and reconstructed coronal (b) plane in soft tissue window reveal a low-density intra-articular mass similar to fat with multiple villi projecting inwards (arrows), which are surrounded by joint fluid with higher density than fat but lower than water. Distention of the joint capsule is evident.

CT revealed a low-density intra-articular mass, similar to fat, with multiple villi, surrounded by joint fluid causing distention of the joint capsule. No bone erosions were seen (Figure 1).

**Figure 2:**
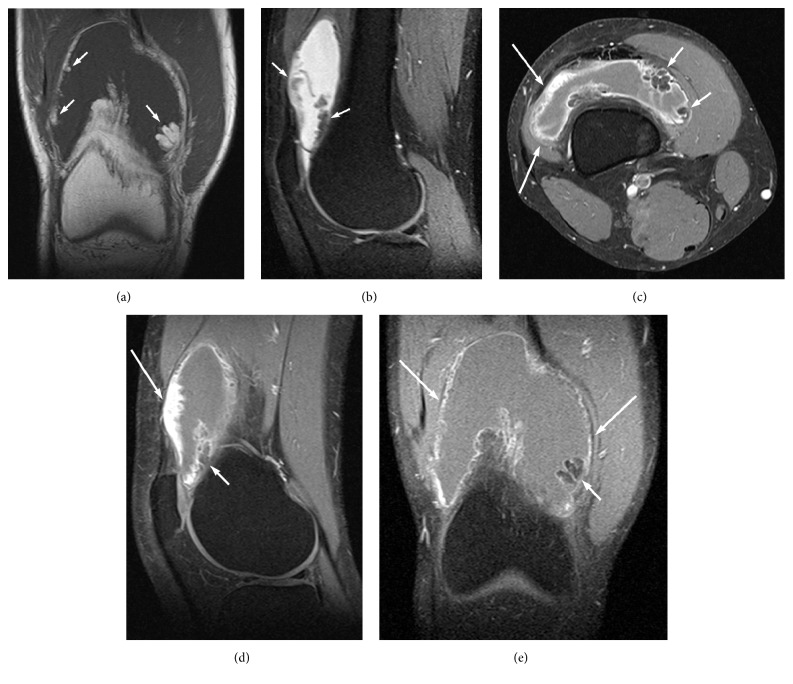
MR images on coronal T1-weighted (a), sagittal PD-weighted with fat saturation (FS) (b), and axial, sagittal, and coronal T1-weighted with FS after iv administration of gadolinium (c), (d), and (e), respectively, depict frond-like villi projecting inwards from the synovium with signal equal to fat on all imaging sequences (short arrows). After iv administration of gadolinium, enhancement of the overlying synovium is observed (long arrows), but no enhancement of the underlying fat is detected. Joint effusion is present distending the joint capsule, without any sign of menisci or ligaments' injury, or osseous erosions.

**Figure 3:**
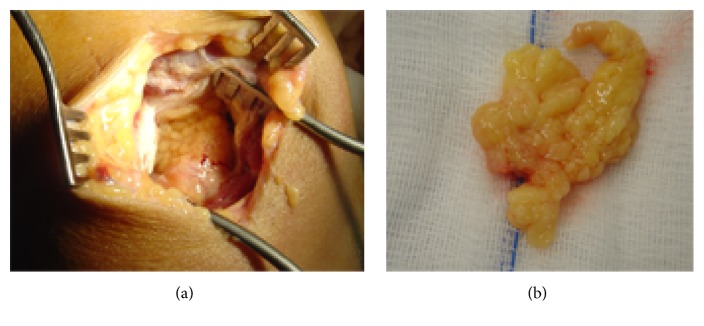
Open biopsy (intraoperative view). Incision is part of the final one (a). Biopsy specimen (b).

MRI depicted joint effusion with multiple, frond-like villi projecting into the joint from the synovium, with signal equal to fat on all imaging sequences. After intravenous (iv) administration of gadolinium, enhancement of the overlying synovium was observed, with no enhancement of the underlying fat (Figure 2). There were no signs of meniscal or ligamentous injury. A probable diagnosis of a LA was considered, based on MRI findings. The patient was programmed for biopsy, which confirmed the diagnosis (Figure 3).

**Figure 4:**
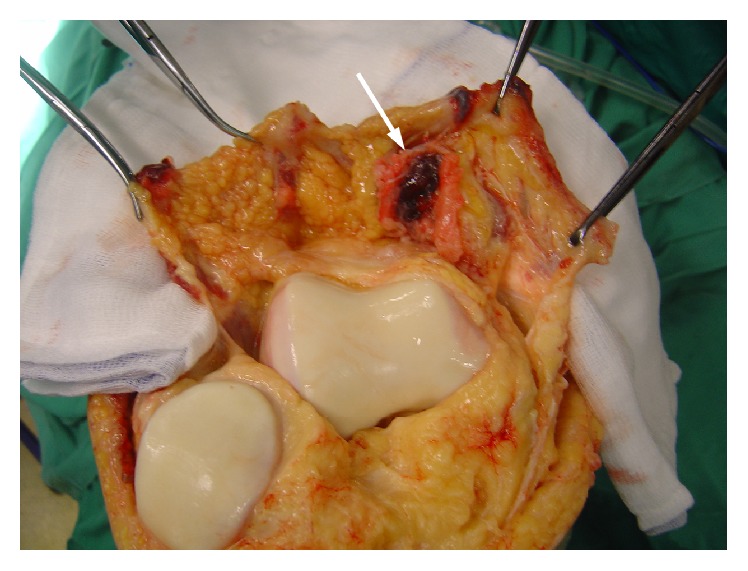
Open synovectomy (intraoperative view). Red-black region of synovium is the site of foregone biopsy (arrow).

An open synovectomy was performed through a medial parapatellar approach, revealing a yellowish villous mass in the suprapatellar pouch. Synovium was meticulously excised from the pouch and the medial gutter. Retropatellar fat pad was debrided as well (Figure 4).

**Figure 5:**
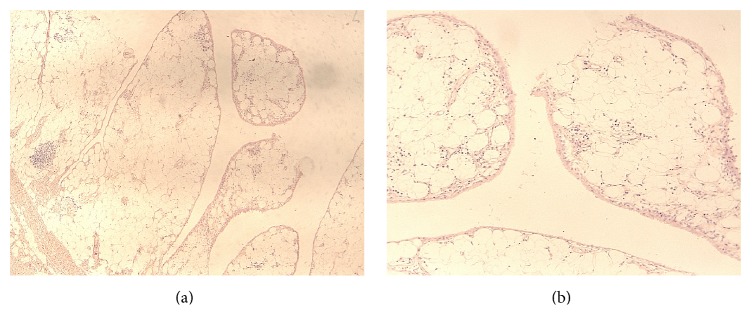
Broad papillary projections lined by synovial membrane. The stroma consists of lobules of mature adipose tissue with focal chronic inflammatory infiltration [Hematoxylin and Eosin stain ×100 (a) and ×200 magnification (b)].

Histological examination verified the diagnosis of LA. It showed broad papillary projections covered by synovium, with lobules of mature adipocytes and focal round-cell inflammatory infiltrations. No signs of malignancy were detected (Figure 5).

**Figure 6:**
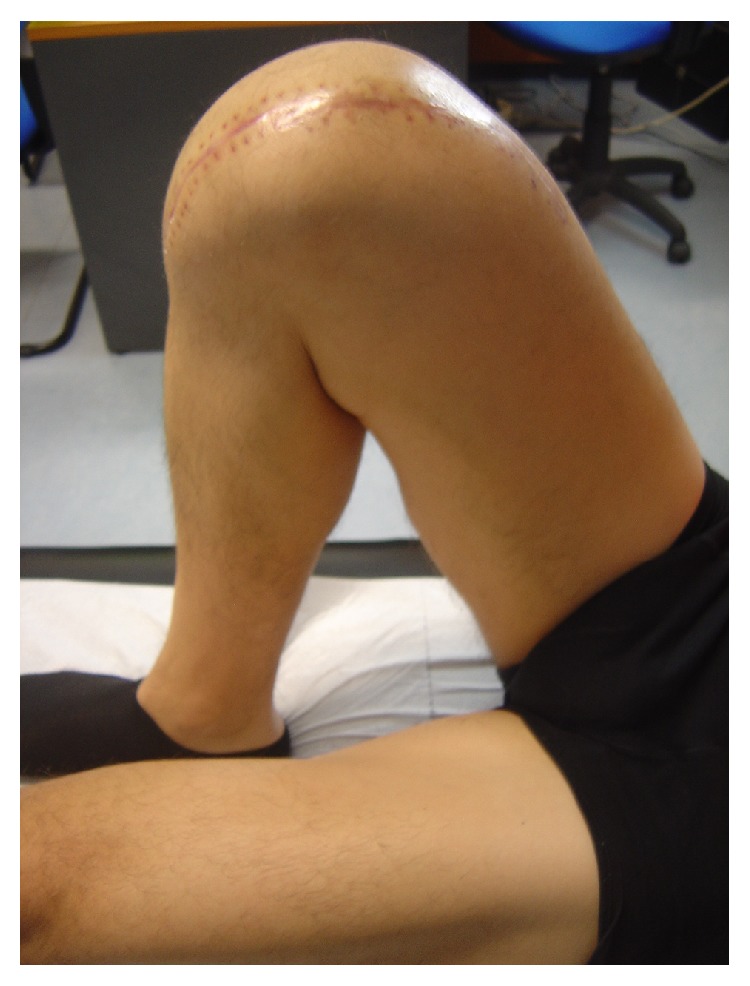
The patient in the first month postoperatively with his operated knee fully flexed.

The patient regained a full range of knee motion from the first postoperative days (Figure 6). Eighteen months postoperatively, the patient has fully returned to his activities and duties without any symptoms.

### 2.2. Patient 2

A 58-year-old male presented with a 4-year history swelling and mild pain in both knees, especially in the right. The patients' previous history was free of trauma, fever, or morning stiffness. No other joints were affected.

Clinical examination showed fullness in the suprapatellar and parapatellar regions of both knees. The swelling was more obvious in the right knee, with tenderness in the medial joint line. The cruciate and collateral ligaments as well as the menisci were intact clinically.

The patient was investigated for rheumatoid factor, serum uric acid, antinuclear antibody, and anti-dsDNA, apart from the routine blood examinations. All findings were normal.

X-rays established the diagnosis of osteoarthritis, with joint space narrowing and osteophytes on both knees, more pronounced on the right.

**Figure 7:**
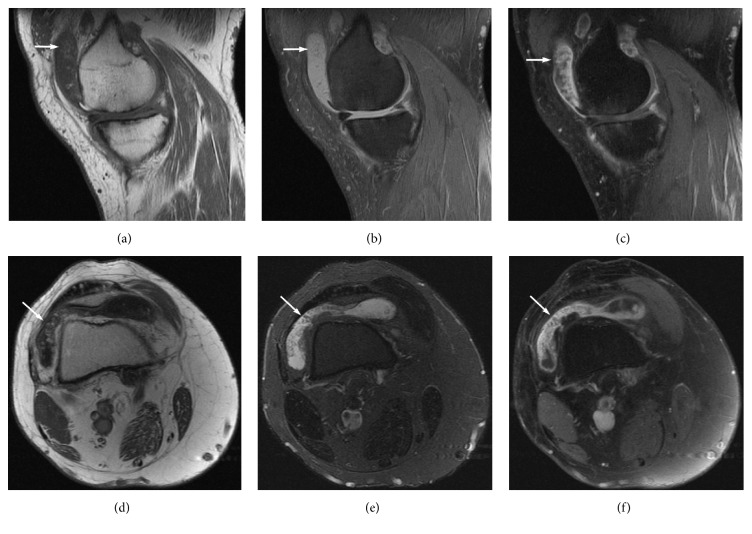
MR images at the same sagittal level on T1-weighted (a), PD-weighted with FS (b), and T1-weighted with FS after iv administration of gadolinium (c) demonstrate medial compartment osteoarthritis with marginal osteophytes, chondral lesions, subchondral changes, and a complex tear of the posterior horn of medial meniscus. Mild villous frond-like fatty subsynovial hyperplasia is present in the suprapatellar bursa associated with synovial proliferation, which is enhanced after iv administration of gadolinium, and joint effusion (arrows). MR images at the same axial level on T1-weighted (d), PD-weighted with FS (e), and T1-weighted with FS after iv administration of gadolinium (f) confirm the above findings (arrows), which are strongly suggestive of LA.

MRI of the right knee demonstrated severe medial compartment osteoarthritis, with marginal osteophytes, chondral lesions, subchondral changes, and a complex tear of the medial meniscus. Mild, villous, frond-like fatty subsynovial hyperplasia was present in the suprapatellar bursa, associated with joint effusion and synovial proliferation, which enhanced after iv administration of gadolinium. These findings were suggestive of LA (Figure 7). MRI of the left knee revealed only degenerative changes, without findings of LA.

Arthroscopic synovectomy of the right knee was performed. Arthroscopic examination of the knee revealed a large amount of yellowish frond-like projections in the suprapatellar pouch, with degenerative changes of the cartilage in the medial and lateral compartment. Synovium was meticulously excised from the suprapatellar pouch and the medial and lateral gutter. Retropatellar fat pad was debrided as well.

Histopathological examination of the tissue revealed fibrous tissue and adipose cells in the subsynovial region, which were responsible for villous dilatation. The villi were covered by hyperplastic inflamed synovium.

The postoperative period was uneventful and the patient returned to his previous level of activity without restriction in the range of movement. There was no recurrence during a twenty-month follow-up period.

### 2.3. Patient 3

A 44-year-old man presented with a 5-year history of pain and swelling in his right knee and feet. He did not report prior trauma or surgery in the affected knee.

Clinical examination disclosed a large amount of knee joint effusion, with restriction of knee movement. Skin examination demonstrated psoriatic plaques and ichthyosis. Blood tests showed C-reactive protein of 10 mg/l and erythrocyte sedimentation rate of 20 mm/h. Antinuclear antibody and anti-dsDNA screen, as well as rheumatoid factor, were negative. The rest of the laboratory findings were unremarkable.

A needle aspiration produced 500 ml of aseptic fluid, containing high numbers of mixed leucocyte populations.

Plain radiographs depicted an area of dense soft tissue in the suprapatellar pouch of the right knee. The femur and tibia were normal, with preservation of the joint space without cortical erosions.

**Figure 8:**
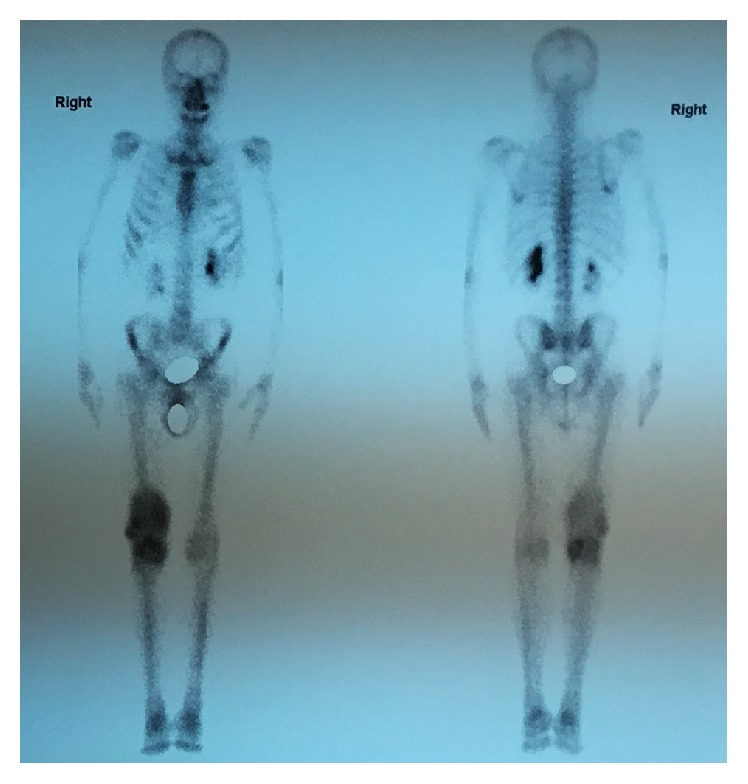
Tc-99m scintigraphy demonstrates persistent concentration of the technetium in the right knee joint, which is indicative of an inflammatory process.

Tc-99m scintigraphy revealed persistent concentration of the technetium in the right knee joint, which was indicative of inflammation. No radionuclide concentration was depicted on the left knee (Figure 8).

**Figure 9:**
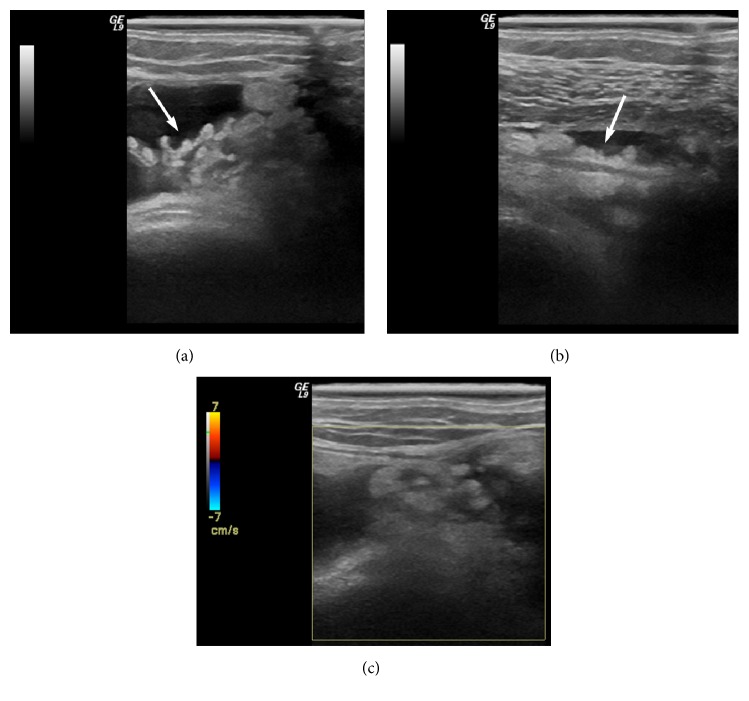
US examination of the right knee on axial (a) and longitudinal (b) sonograms reveals hyperechoic frond-like projections of the synovium into the effusion (arrows), findings suggestive of LA. Colour Doppler US (c) does not depict any blood flow in the affected area.

Ultrasonographic examination revealed hyperechoic frond-like projections of the synovium into the effusion (Figure 9).

**Figure 10:**
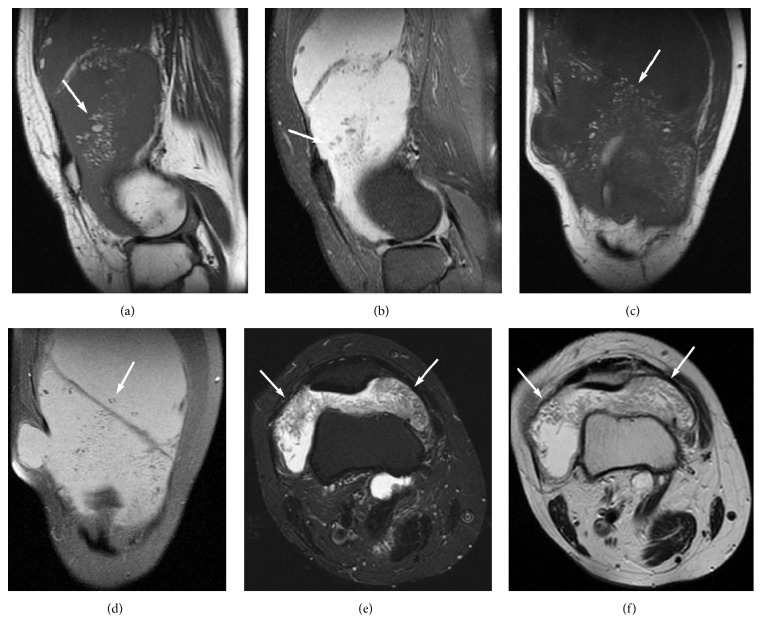
MR images on sagittal T1-weighted (a) and PD-weighted with FS (b), on coronal T1-weighted (c) and PD-weighted with FS (d), and on axial STIR (e) and T2-weighted (f) demonstrate synovial proliferation and a large primarily fat-signal mass on all MR sequences, filling much of the suprapatellar bursa with a frond-like appearance, which is surrounded by excessive joint fluid. The innumerable individual villi are small in this case, with small quantities of interposed fluid, creating a feathery appearance (arrows). These findings are indicative of a large feathery-appearing LA filling the suprapatellar recess.

MRI of the right knee demonstrated synovial proliferation and a large, primarily fat-signal mass, on all MR sequences, filling much of the suprapatellar bursa, with a frond-like appearance, surrounded by excessive joint fluid. The innumerable individual villi were small in this case, with small quantities of interposed fluid, creating a feathery appearance. These findings were strongly suggestive of LA (Figure 10). MRI of the left knee was normal.

The patient was treated by open synovectomy of the affected knee. A yellowish villous mass was removed from the suprapatellar pouch and the medial and lateral gutter. Synovium was carefully excised from the pouch, the retropatellar fat pad, and Hoffa's fat pad.

Histological assessment of LA tissue disclosed frondose areas with significant neovascularization, dense cellular infiltrates, and connective tissue with mature adipocytes.

The postoperative period was uneventful, and the patient returned to his previous level of activity with improvement in the range of knee flexion. There was no recurrence up to 18 months after surgery.

## 3. Discussion

LA is an unusual, intra-articular, benign lesion of synovium. The first detailed case report was by Arzimanoglu in 1957 [4]. Since then, less than 100 have been reported according to Kamaci et al. [5]. Most reports consist of just one case or a small series of this unusual lipoma, while Howe and Wenger described the largest series with 45 lesions in 39 patients [6].

Its etiology and pathogenesis are unknown. A history of trauma is reported in many patients with LA [7, 8]. Diabetes mellitus or steroid use have also been described [6]. Lesions due to either degenerative or inflammatory arthritis are seen quite often, as well [6, 9–11]. Indeed, Howe and Wenger reported the presence of arthritic chondral damage at 36 of their 39 patients [6]. Moreover, late treatment is related to early development of osteoarthritis [12].

According to Kloen et al., a typical patient with LA is an adult in the fourth or fifth decade of his life [1]. Men and women are equally affected. Howe and Wenger describe unilateral knee involvement as typical, while atypical cases include both knees or involvement of other joints, such as shoulder, elbow, wrist, hip and ankle [6, 7, 13–16]. Patients complain of chronic, progressive, painless swelling of the involved joint. Effusion is almost always present, but limitations in range of movement and pain are not seen very often [1, 13, 17].

Blood tests are normal and plain radiographs may show soft tissue density in the suprapatellar pouch when the knee is involved [17, 18].

MRI is the diagnostic imaging modality of choice and can demonstrate variable morphological patterns with pathognomonic characteristics [1, 19]. A large frond-like mass arising from the synovium is seen, with signal intensity similar to fat on all pulse sequences [20–22]. Alternatively, multiple villous proliferation of the synovium and fatty-appearing globules can be seen, while a mixed pattern can also appear [22]. Although the subsynovial tissue does not enhance after iv administration of contrast medium, the synovial lining and the joint fluid may demonstrate enhancement, related to the presence of inflammatory cells [10]. Finally, the absence of magnetic susceptibility artifacts attributable to hemosiderin is also characteristic and useful in the differential diagnosis [19].

Although LA has characteristic features that usually make it a straightforward MRI diagnosis, other entities presenting as filling defects in the suprapatellar recess, such that osseous loose bodies, synovial chondromatosis, “rice bodies” accompanying rheumatoid arthritis, pigmented villonodular synovitis, quadriceps fat pad impingement, synovial hemangioma, intra-articular lipoma, and intra-articular liposarcoma may mimic LA and cause confusion [23, 24].

Macroscopically, LA appears as a yellowish synovial proliferation, with broad villous projections of fatty tissue arranged in an arborescent pattern, usually filling the suprapatellar pouch and both gutters [18, 25]. Histologically, massive infiltration of the synovium by mature adipose cells is characteristic for LA. Enlarged hyperaemic capillaries and focal chronic inflammatory infiltration is usually observed [18].

Although our first patient is a typical LA patient regarding age and MR imaging findings, his symptoms and clinical signs at time of presentation were quite atypical. A painful knee swelling is not so common in patients with LA. It is also very unusual that our patient's knee was becoming less painful as swelling was progressing, a characteristic that has not been mentioned in other painful LAs. Moreover, night knee pain that awakens the patient has not been described in the literature so far being more commonly associated with bone sarcomas with an incidence ranging from 20 to 44% and may mislead to false diagnosis [26, 27].

The third case presented here is extremely rare and refers to unilateral LA in a patient with psoriatic arthritis. To the best of our knowledge, there are only two cases reported in the literature referring to concurrent psoriatic arthritis with LA [28, 29]. However, in our patient, the MRI appearance is very unusual, with innumerable individual villi and with small quantities of interposed fluid, creating a feathery appearance. Areas of hypertrophic synovium with little or no fat deposition were also present. When LA develops in patients with chronic synovitis, as in this patient with psoriatic arthritis, areas of synovial thickening without fat deposition are often encountered.

LA is a benign tumor. Malignancy has not been reported, so biopsy is not regarded by some authors as an essential part of treatment algorithm [1, 25, 30, 31]. However, considering the small number of cases with LA that have been described in the literature so far, as well as the fact that the pathology of this entity has not been clarified up till now, we believe that biopsy may be useful in selected cases. Recommended treatment is open synovectomy and recurrence after surgery is uncommon [11, 30]. Arthroscopic synovectomy is also described in recent case reports, with comparable rates of recurrence [25, 30, 32]. Chemical synovectomy with Yttrium has been used for temporal relief of the symptoms, but not as the definitive treatment [18].

When synovectomy is delayed more than a year from symptoms onset, early osteoarthritis may develop [12]. One of the largest case series showed osteoarthritic signs two years postoperatively in all patients treated with a delay [12].

Finally, although MRI with iv administration of gadolinium is extremely useful diagnostically, the definite diagnosis can only be established by histological examination, which reveals diffuse replacement of the subsynovial tissue with mature fat cells, causing villous expansion of the synovium, and inflammatory cells around capillaries [12, 33].

In conclusion, LA is a relatively uncommon benign lesion, which typically affects knee joint, especially the suprapatellar pouch. It should always be considered in the spectrum of differential diagnosis when clinicians deal with chronic swelling, either painful or painless, of a joint. MRI findings are characteristic and early synovectomy offers the best functional outcome.
